# Effects of Spatial Frequencies on Word Identification by Fast and Slow Readers: Evidence from Eye Movements

**DOI:** 10.3389/fpsyg.2016.01433

**Published:** 2016-09-28

**Authors:** Timothy R. Jordan, Jasmine Dixon, Victoria A. McGowan, Stoyan Kurtev, Kevin B. Paterson

**Affiliations:** ^1^Department of Psychology, Zayed UniversityDubai, United Arab Emirates; ^2^Department of Neuroscience, Psychology, and Behaviour, University of LeicesterLeicester, UK

**Keywords:** eye movements during reading, spatial frequencies, reading comprehension, reading, word recognition, reading ability, reading speed

## Abstract

Recent research has shown that differences in the effectiveness of spatial frequencies for fast and slow skilled adult readers may be an important component of differences in reading ability in the skilled adult reading population (Jordan et al., 2016a). But the precise nature of this influence on lexical processing during reading remains to be fully determined. Accordingly, to gain more insight into the use of spatial frequencies by skilled adult readers with fast and slow reading abilities, the present study looked at effects of spatial frequencies on the processing of specific target words in sentences. These target words were of either high or low lexical frequency and each sentence was displayed as normal or filtered to contain only very low, low, medium, high, or very high spatial frequencies. Eye movement behavior for target words was closest to normal for each reading ability when text was shown in medium or higher spatial frequency displays, although reading occurred for all spatial frequencies. Moreover, typical word frequency effects (the processing advantage for words with higher lexical frequencies) were observed for each reading ability across a broad range of spatial frequencies, indicating that many different spatial frequencies provide access to lexical representations during textual reading for both fast and slow skilled adult readers. Crucially, however, target word fixations were fewer and shorter for fast readers than for slow readers for all display types, and this advantage for fast readers appeared to be similar for normal, medium, high, and very high spatial frequencies but larger for low and very low spatial frequencies. Therefore, although fast and slow skilled adult readers can both use a broad range of spatial frequencies when reading, fast readers make more effective use of these spatial frequencies, and especially those that are lower, when processing the identities of words.

## Introduction

During reading, the eyes move along lines of text in a series of saccadic movements, each movement ending in a brief fixational pause (Rayner, 1998, 2009). During these pauses, visual information is acquired from the text and the number and duration of the fixations made are highly informative about the process of word identification that takes place during reading. In particular, the forward movement of the eyes through text appears to be driven by processes underlying the identification of words, and findings show that words with a lower frequency of written usage, which are less familiar, are fixated for longer (e.g., Inhoff and Rayner, 1986; Rayner and Duffy, 1986; Rayner et al., 1996; Juhasz and Rayner, 2003; Juhasz et al., 2006; Paterson and Jordan, 2010). Such findings are central to cognitive control theories of eye movement behavior (e.g., Rayner et al., 1996), and have been fundamental to the development of formal computational models of eye movement control (e.g., Reichle et al., 1998, 2003; Engbert et al., 2005). However, the nature of the visual information acquired on each fixation, and its effectiveness for supporting access to lexical representations, is not yet well-established.

It is of particular relevance for the present research that visual pathways in the brain are selectively sensitive to spatial frequencies associated with different scales of visual information (e.g., Robson, 1966; Blakemore and Campbell, 1969; Lovegrove et al., 1980). In this sense, the term spatial frequency refers to the level of detail present in a stimulus, per degree of visual angle. So, for example, a scene with small detailed objects is likely to contain more high spatial frequency information than one composed of large coarse objects, and the spatial frequencies contained in such scenes provide the visual bases for recognizing the objects that are present. In a similar vein, the visual system acquires a broad range of different spatial frequencies from text and these provide the fundamental bases for subsequent linguistic analyses that enable readers to identify the words that are present. For instance, lower spatial frequencies may allow readers to establish a word's overall shape and location but not the fine detail of its component letters. By comparison, higher spatial frequencies may enable readers to see a word's fine detail, such as the precise form and location of individual letters, but are less useful for perceiving the overall shapes of words (e.g., Legge et al., 1985; Jordan, 1990, 1995; Patching and Jordan, 2005a,b; Allen et al., 2009; Kwon and Legge, 2012). Consequently, although the influence of spatial frequencies is not likely to be apparent to readers, processes that underlie the identification of words rely fundamentally on these low-level visual properties. However, the effectiveness of different spatial frequencies for accessing lexical representations and how this might differ for readers of different abilities remains unclear.

While skilled adult reading generally is fast (up to 400–500 words per minute), substantial variation in reading speed exists across the skilled adult reading population (e.g., Jackson and McClelland, 1979; Andrews, 2008, 2012; Rayner et al., 2010; Ashby et al., 2012). This may be due to faster readers making greater use of predictive processes to anticipate the identities of words, based on the parafoveal processing of upcoming text or contextual expectations (e.g., Long et al., 1994; Murray and Burke, 2003; Hersch and Andrews, 2012; Frömer et al., 2015; Hawelka et al., 2015). However, others have argued that fast reading is facilitated by “bottom-up” processes that enable readers to gain visual access to lexical representations and so identify words more rapidly. For instance, the lexical quality hypothesis of reading skill (e.g., Perfetti, 1992, 2007; see also Andrews, 2008, 2012; Veldre and Andrews, 2014) proposes that fast readers have high-quality lexical representations in which orthographic information that defines a particular word is stored precisely, so that a written word can activate its correct lexical representation directly from the visual input. By comparison, slow readers have underspecified lexical representations such that the stored orthographic information defining a particular word is imprecise and rapid bottom-up reading is prevented (e.g., Perfetti, 1992, 2007; Andrews, 2008, 2012).

Consistent with this general view, skilled readers provide quicker responses than less skilled readers in tasks requiring the recognition of single words (e.g., Andrews and Hersch, 2010; Andrews and Lo, 2012) and, during textual reading, skilled readers can identify more quickly than less skilled readers unfamiliar words that are highly predictable from prior context (Ashby et al., 2005). Moreover, recent research with adolescent readers shows that fast readers make fewer and shorter fixational pauses on words, longer forward saccades, and fewer regressions (i.e., backward saccades) than slow readers (Krieber et al., 2016). However, the nature of the visual input that contributes to fast and slow reading abilities is not completely clear. In particular, research to date has focused on the role of letter identities and letter positions in this input (e.g., Perfetti, 1992, 2007; Andrews, 2008, 2012). But as the fundamental visual components of words are spatial frequencies, not letters, other researchers have argued that access to lexical representations may be achieved by a broad range of spatial frequencies and even by relatively coarse-scale information provided by lower spatial frequency input (e.g., Jordan, 1990, 1995; Patching and Jordan, 2005a,b; Allen et al., 2009). Indeed, whereas high spatial frequencies are conveyed relatively slowly by parvocellular pathways in the brain, low spatial frequencies are carried by fast magnocellular pathways and so may provide especially rapid access to lexical entries (for a review, see Hegdé, 2008). It is therefore important to establish if differences in the use of high and low spatial frequencies by fast and slow readers underlie a word recognition advantage for fast readers.

While spatial frequency sensitivity by dyslexic and non-dyslexic readers has been widely investigated (for a review, see Skottun, 2000), the effectiveness of the spatial frequency content of text for fast and slow skilled adult word recognition has yet to be fully revealed. An especially relevant study by Patching and Jordan (2005a) showed that briefly-presented words, filtered to contain only certain spatial frequencies, can be recognized equally accurately by fast and slow readers. However, the recognition of brief single words is not the same as textual reading and research shows that eye movements during textual reading are highly informative about individual differences in reading performance (e.g., Ashby et al., 2005; Rayner, 2009; Rayner et al., 2010; Kuperman and Van Dyke, 2011). Indeed, other investigations show that when lines of text are filtered so that only certain spatial frequencies remain, readers use a broad range of different spatial frequencies, and these different spatial frequencies produce different patterns of eye movement behavior (Jordan et al., 2012, 2014; Paterson et al., 2012, 2013a,b). However, this research did not examine whether these influences of spatial frequencies on textual reading differ for fast and slow reading abilities.

**Figure 1:**
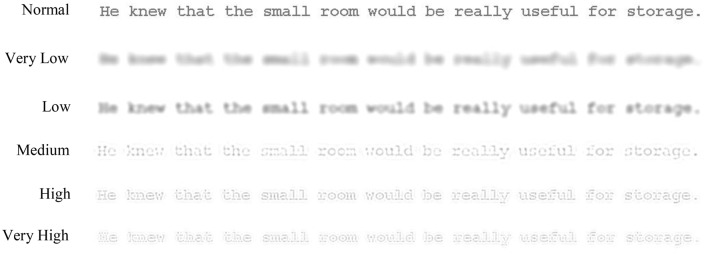
**Examples of the types of display used in the experiment**. The figure shows a sentence displayed as normal and filtered to contain only very low, low, medium, high, and very high spatial frequencies. The visual appearance of the filtered displays shown in the figure is approximate due to variations in display resolution and print medium.

A very recent study (Jordan et al., 2016a) examined this issue in more detail by comparing the eye movements of fast and slow skilled adult readers when sentences were presented either as normal or filtered to contain only certain bands of spatial frequencies ranging from very low to very high (see Figure 1). The findings for normal text replicated reading time effects for fast and slow readers in other research (e.g., Krieber et al., 2016), but in addition showed that a broad range of spatial frequencies can support normal reading for both fast and slow readers. However, while reading times for normal text and for each spatial frequency were all shorter for fast readers than for slow, the advantage for fast readers was similar to normal for medium and higher spatial frequencies but substantially larger for lower spatial frequencies. The indication from this work, therefore, is that while fast and slow readers can both use a range of different spatial frequencies for reading, fast readers make more effective use of the spatial frequencies in text and especially those that are lower.

But this previous study focused on reading times and eye movements averaged across whole sentences, and so it remains to be confirmed that the overall advantage observed for fast readers reflects an ability to actually obtain more rapid access to lexical representations. Clearly, gaining a better understanding of the use of spatial frequencies by fast and slow readers when processing the identities of individual words in text is crucial for revealing the role of spatial frequencies in reading and is especially relevant to accounts of reading ability that propose that fast reading benefits from rapid visual access to lexical representations (e.g., Perfetti, 1992, 2007; Andrews, 2008, 2012). Accordingly, to achieve this, we revisited our earlier experiment (Jordan et al., 2016a) in order to investigate effects of spatial frequencies on individual word recognition. One aspect of this experiment that is so far unexamined is that the stimuli used were from a stimulus set in which sentences contained matched target words of high and low lexical frequency and so this would allow the investigation of lexical frequency effects during reading. Consequently, while this aspect of that experiment was neither inspected nor reported by Jordan et al. (2016a) these additional data have the capacity to shed important light on the use of spatial frequencies by fast and slow skilled adult readers when processing the identities of individual words during reading.

If fast reading relies on detailed analyses of orthographic content (as the lexical quality hypothesis suggests), the processing of words in filtered displays may show an advantage for fast readers that is maximal when spatial frequencies provide this detail (i.e., in displays containing medium and higher spatial frequencies) and this advantage may be reduced (or even be absent) when displays contain only lower spatial frequencies. The manipulation of lexical frequency also allowed a closer look at the effectiveness of different spatial frequencies for supporting the identification of high and low lexical frequency words during reading. High lexical frequency words typically receive fewer and shorter fixations than low lexical frequency words when reading, and this lexical frequency effect is generally taken as a hallmark of normal processes of word identification (e.g., Inhoff and Rayner, 1986; Rayner and Duffy, 1986; Rayner et al., 1996; Juhasz and Rayner, 2003; Juhasz et al., 2006; Paterson and Jordan, 2010). Consequently, if spatial frequencies support normal processes of word identification, lexical frequency effects should be observed when these spatial frequencies are present in text. Word frequency effects are also usually larger when word identification is more difficult, due to readers experiencing disproportionately greater difficulty recognizing lower lexical frequency words (see, e.g., Rayner et al., 1998; Paterson and Jordan, 2010). Consequently, spatial frequencies that are less effective at supporting word identification may produce atypically-large effects of lexical frequency. However, spatial frequencies that provide little or no support to word identification may more seriously impair the normal processing of text so that standard lexical frequency effects are no longer observed. Finally, if fast readers benefit more than slow readers from the presence of particular spatial frequencies, lexical frequency effects may differ for fast and slow reading abilities depending on which spatial frequencies are present in text.

## Methods

### Participants

This research was conducted with the ethical approval of the Research Ethics Committee at the University of Leicester, and in accordance with the principles expressed in the Declaration of Helsinki. All participants gave informed consent in writing. Thirty participants (aged 18–30 years) from the University of Leicester were screened for reading speed prior to participating in the experiment. All were native English speakers and had normal or corrected vision, as determined by Bailey-Lovie (Bailey and Lovie, 1980), ETDRS (Ferris and Bailey, 1996), and Pelli-Robson (Pelli et al., 1988) assessments (see Jordan et al., 2011). Vocabulary was, in addition, assessed using the Nelson-Denny vocabulary test (Brown et al., 1993).

Following previous research (e.g., Jackson and McClelland, 1979; Patching and Jordan, 2005a,b; see also Rayner et al., 2010), fast and slow readers were identified by their effective reading speed, which was calculated for each participant by multiplying reading speed in words per minute (wpm) by the proportion of reading comprehension questions answered correctly. This was achieved using 4 passages (mean length = 526 words) presented on a high-definition display. Participants read all 4 passages normally and each was followed by 6 multiple-choice questions that assessed comprehension. Effective reading speeds ranged from 226 to 443. Fast readers were classified as the upper 50% of this range (15 participants) and slow readers were the lower 50% (15 participants). Effective reading speeds were 325–443 for the fast readers and 226–318 for the slow readers. Slow readers read test passages significantly more slowly than fast readers, *t*_(28)_ = 2.65, *p* < 0.01, *r* = 0.44, had lower comprehension accuracy, *t*_(28)_ = 3.55, *p* < 0.01, *r* = 0.54, and scored lower on the Nelson-Denny vocabulary test than fast readers [*t*_(28)_ = 3.33, *p* < 0.01, *r* = 0.52]. But fast and slow readers did not differ in performance on any tests of visual abilities (all *t*s < 1.3; Table 1).

**Table 1:** **Visual and vocabulary performance of fast and slow readers**.

**Reading ability**	**High contrast acuity (near)**	**High contrast acuity (distant)**	**Low contrast acuity (near)**	**Low contrast acuity (distant)**	**Contrast sensitivity**	**Vocabulary (%)**
Fast	20/20.6	20/21.2	20/26.6	20/32.8	1.95	92
Slow	20/20.4	20/23.4	20/27.9	20/33.2	1.95	80

### Stimuli and design

Stimuli were 120 standardized sentences (see McGowan et al., 2015) that included either a high or low lexical frequency target word located toward the middle of each sentence and which had previously shown robust lexical frequency effects when sentences were presented as normal. High and low lexical frequency members of each target word pair were matched for length (*M* = 5.3 letters, range = 4–6 letters) and occupied the same location within their shared sentence. Sentence contexts were chosen so that each high/low lexical frequency word pair was equally plausible within the sentence context and equally unpredictable, as evidenced by prescreening (see McGowan et al., 2015). High lexical frequency words had a mean Zipf frequency of 5.24 and low lexical frequency words a mean Zipf frequency of 3.36 (according to the SUBTLEX-UK database; van Heuven et al., 2014) corresponding to actual mean frequencies of occurrence of 249.1 and 2.3 per million, respectively.

Each sentence context and high/low lexical frequency target word combination was displayed in 1 of 6 display conditions, where each sentence was shown entirely as normal or filtered to leave one of 5 different, 1-octave wide bands of spatial frequencies with low- and high-pass cut-off frequencies of 1.65–3.3, 2.6–5.2, 5.0–10.0, 8.3–16.6, and 10.3–20.6 cycles per degree (see Figure 1). These 5 bands were termed very low, low, medium, high, and very high. The sentences were displayed to participants so that each participant viewed an equal number of sentences containing high and low lexical frequency target words in each of the 6 display conditions but each participant viewed only one version of each sentence (with either its high or low lexical frequency target), and each sentence was shown equally often with its high or low lexical frequency target in each display condition over the experiment. Sentences were shown to each participant in a randomized order. Additional sentences (2 per display condition) were used as practice items at the start of each session.

### Apparatus and procedure

Eye movements were recorded using an Eyelink 1000 tower-mounted eye-tracker with chin and forehead rest. Viewing was binocular and each participant's right eye movements were sampled at 1000 Hz using pupil-tracking and corneal reflection. Sentences were displayed on a high-definition 19-inch monitor and a 4-letter word subtended approximately 1° (i.e., normal size for reading; Rayner and Pollatsek, 1989). The eye-tracker was calibrated at the beginning of the experiment. On each trial, participants fixated a cross presented on the left of the screen, and a sentence was then presented, with its first letter replacing this cross. Participants were instructed to read normally and for comprehension and answered a comprehension question after each sentence.

## Results

All participants produced high levels of overall accuracy for the sentence comprehension questions (mean = 91% correct) and no differences in comprehension were observed between reading abilities for any display type, *F* < 1.1. Differences were observed, however, across the displays, *F*_(5, 140)_ = 67.88, *p* < 0.001, $\eta_{p}^{2}=$ 0.71, and comprehension accuracy was equally high for text shown normally (95%) and filtered to contain only low (93%), medium (96%), high (95%), or very high (93%) spatial frequencies, but lower for very low spatial frequencies (61%; *p*s < 0.01).

**Figure 2:**
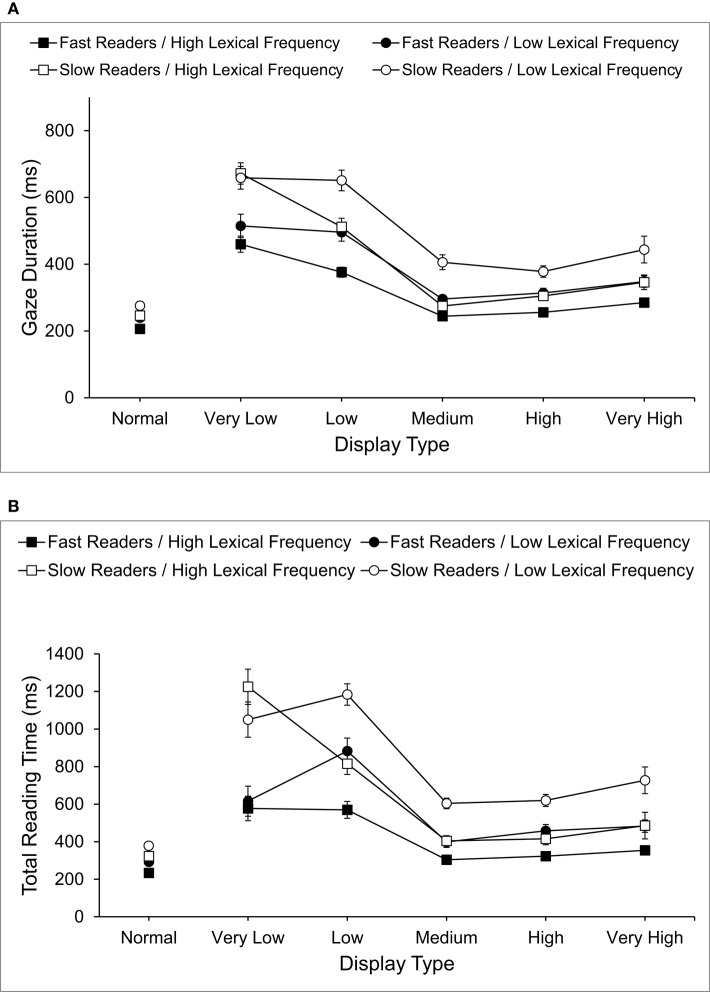
**(A)** Mean Gaze Durations and **(B)** mean Total Reading Times (including standard error bars) for fast and slow readers.

The focus of the present investigation was the influence of normal and filtered displays on eye movements for target words in sentences and so the following standard word-level measures of eye movements are reported (see Rayner, 2009): word-skipping (the probability of a target word not receiving a fixation during first-pass reading), first-pass fixations (the number of fixations on a target word during first-pass reading), first-fixation duration (the duration of the first, first-pass fixation on a target word), single-fixation duration (the duration of the first fixation for target words that receive only one first-pass fixation), gaze duration (the sum of all first-pass fixation durations prior to a fixation on another word), total reading time (the sum of all fixation durations on a target word), regressions out (the probability of a regressive saccade away from a target word), and regressions in (the probability of a regressive saccade to a target word). Mean eye movement measures for high and low lexical frequency target words in each display condition for each reading ability are shown in Table 2 and gaze durations and total reading times for target words are shown graphically in Figure 2. Data for each measure were analyzed using Analysis of Variance with factors reading ability (fast, slow), display condition (normal, very low, low, medium, high, very high), and target word lexical frequency (high, low), with error computed across participants (*F*_1_) and word stimuli (*F*_2_). Additional pairwise comparisons were performed using Bonferroni corrections.

**Table 2:** **Mean eye movement measures for fast and slow readers in each display and target word lexical frequency condition**.

	**Display type**												
	**Reading ability**	**Normal**		**Very low**		**Low**		**Medium**		**High**		**Very high**	
		**Target word lexical frequency**											
		**High**	**Low**	**High**	**Low**	**High**	**Low**	**High**	**Low**	**High**	**Low**	**High**	**Low**
Word skipping (%)	Fast	16.7 (2.5)	10.7 (1.8)	27.3 (6.0)	24.7 (6.2)	7.3 (1.3)	3.3 (0.9)	12.0 (2.1)	7.3 (1.6)	6.7 (2.0)	4.7 (1.2)	8.7 (1.8)	2.7 1.1)
	Slow	13.3 (1.8)	8.9 (2.2)	8.0 (2.3)	10.9 (2.4)	2.0 (.8)	4.9 (1.4)	8.7 (2.2)	4.9 (1.7)	6.0 (1.5)	3.8 (1.6)	4.7 (1.2)	3.8 (1.6)
First-fixation duration (ms)	Fast	200 (3)	218 (5)	370 (17)	371 (17)	291 (9)	330 (12)	228 (6)	249 (6)	237 (6)	255 (9)	251 (9)	277 (8)
	Slow	241 (8)	244 (7)	402 (14)	390 (16)	306 (16)	315 (16)	259 (6)	259 (10)	258 (9)	262 (10)	276 (12)	279 (10)
Single-fixation duration (ms)	Fast	201 (3)	220 (5)	390 (20)	401 (23)	304 (10)	340 (15)	229 (6)	256 (7)	242 (6)	261 (8)	258 (11)	292 (8)
	Slow	233 (9)	249 (7)	500 (17)	485 (23)	380 (20)	380 (19)	256 (7)	313 (12)	260 (10)	299 (12)	285 (12)	326 (11)
Gaze duration (ms)	Fast	206 (3)	240 (7)	460 (25)	515 (35)	376 (15)	496 (27)	244 (6)	295 (11)	256 (7)	314 (14)	285 (11)	348 (17)
	Slow	246 (9)	276 (12)	672 (32)	659 (34)	512 (26)	651 (31)	275 (8)	406 (22)	305 (14)	378 (17)	345 (22)	444 (40)
Number of first-pass fixations	Fast	1.0 (0.01)	1.1 (0.02)	1.3 (0.05)	1.3 (0.08)	1.3 (0.05)	1.5 (0.06)	1.1 (0.02)	1.2 (0.04)	1.1 (0.01)	1.2 (0.03)	1.1 (0.02)	1.3 (0.03)
	Slow	1.1 (0.02)	1.1 (0.04)	1.6 (0.06)	1.6 (0.05)	1.5 (0.04)	1.7 (0.06)	1.1 (0.02)	1.4 (0.07)	1.2 (0.05)	1.4 (0.03)	1.2 (0.05)	1.4 (0.11)
Total reading time (ms)	Fast	234 (12)	293 (19)	577 (65)	616 (80)	570 (45)	883 (69)	304 (22)	399 (29)	323 (19)	458 (34)	354 (22)	483 (33)
	Slow	323 (14)	378 (16)	1225 (118)	1050 (94)	815 (40)	1184 (57)	404 (21)	604 (28)	416 (16)	620 (32)	486 (26)	727 (71)
Regressions-out (%)	Fast	9.6 (1.7)	4.7 (1.4)	14.2 (2.7)	10.0 (2.5)	14.8 (3.6)	15.7 (3.3)	5.6 (1.4)	8.0 (1.9)	8.9 (2.1)	7.3 (1.6)	7.0 (2.2)	5.3 (1.7)
	Slow	27.7 (3.4)	31.9 (3.4)	44.6 (3.2)	32.0 (4.0)	35.8 (2.7)	39.9 (3.9)	22.7 (3.0)	38.3 (3.4)	25.9 (2.7)	33.3 (1.8)	24.8 (2.9)	34.7 (1.8)
Regressions-in (%)	Fast	18.8 (2.7)	24.1 (2.5)	19.5 (3.3)	21.8 (3.8)	29.4 (2.8)	37.9 (3.6)	20.1 (3.3)	25.8 (3.8)	19.3 (3.1)	29.5 (4.0)	21.7 (2.9)	28.0 (3.4)
	Slow	27.7 (3.4)	31.9 (3.4)	44.6 (3.3)	32.0 (4.0)	35.8 (2.7)	37.8 (3.9)	22.7 (3.0)	38.3 (3.4)	25.9 (2.7)	33.3 (1.8)	24.8 (2.9)	34.7 (3.0)

Evidence of main effects of reading ability was obtained for all measures [word-skipping, *F*_1(1, 28)_ = 6.19, *p* < 0.02, $\eta_{p}^{2}$ = 0.18, *F*_2(1, 118)_ = 19.07, *p* < 0.001, $\eta_{p}^{2}=$ 0.14; first-pass fixations, *F*_1(1, 28)_ = 14.25, *p* < 0.01, $\eta_{p}^{2}=$ 0.34, *F*_2(1, 118)_ = 36.82, *p* < 0.001, $\eta_{p}^{2}$ = 0.28; first-fixation durations, *F*_1(1, 28)_ = 10.46, *p* < 0.01, $\eta_{p}^{2}=$ 0.28, *F*_2(1, 118)_ = 63.91, *p* < 0.001, $\eta_{p}^{2}=$ 0.41; single-fixation durations, *F*_1(1, 28)_ = 8.62, *p* < 0.01, $\eta_{p}^{2}=$ 0.24, *F*_2(1, 118)_ = 42.64, *p* < 0.001, $\eta_{p}^{2}=$ 0.54; gaze durations, *F*_1(1, 28)_ = 23.05, *p* < 0.001, $\eta_{p}^{2}=$ 0.46, *F*_2(1, 118)_ = 93.63, *p* < 0.001, $\eta_{p}^{2}=$ 0.50; total reading times, *F*_1(1, 28)_ = 28.64, *p* < 0.001, $\eta_{p}^{2}=$ 0.51, *F*_2(1, 118)_ = 280.94, *p* < 0.001, $\eta_{p}^{2}=$ 0.70; regressions in, *F*_1(1, 28)_ = 4.61, *p* < 0.05, $\eta_{p}^{2}=$ 0.14, *F*_2(1, 118)_ = 37.55, *p* < 0.001, $\eta_{p}^{2}=$ 0.24; regressions out, *F*_1(1, 28)_ = 71.96, *p* < 0.001, $\eta_{p}^{2}=$ 0.72, *F*_2(1, 118)_ = 11.38, *p* < 0.01, $\eta_{p}^{2}=$ 0.09]. Compared to fast readers, slow readers skipped words less frequently, made more and longer fixations, and more regressions (word-skipping, slow readers = 6.2%, fast readers = 11.0%; first-pass fixations, slow readers = 1.4, fast readers = 1.2; first-fixation durations, slow readers = 319 ms, fast readers = 273 ms; single-fixation durations, slow readers = 331 ms, fast readers = 283 ms; gaze durations, slow readers = 431 ms, fast readers = 336 ms; total reading times, slow readers = 686 ms, fast readers = 458 ms; regressions in, slow readers = 32.5%, fast readers = 24.7%; regressions out, slow readers = 32.5%, fast readers = 9.3%).

Evidence of main effects of display condition was also obtained for all measures [word-skipping, *F*_1(5, 140)_ = 6.50, *p* < 0.001, $\eta_{p}^{2}=$ 0.19, *F*_2(5, 590)_ = 18.71, *p* < 0.001, $\eta_{p}^{2}=$ 0.14; first-pass fixations, *F*_1(5, 140)_ = 13.75, *p* < 0.001, $\eta_{p}^{2}=$ 0.33, *F*_2(5, 590)_ = 22.55, *p* < 0.001, $\eta_{p}^{2}=$ 0.19; first-fixation durations, *F*_1(5, 140)_ = 89.97, *p* < 0.001, $\eta_{p}^{2}=$ 0.77, *F*_2(5, 590)_ = 131.70, *p* < 0.001, $\eta_{p}^{2}=$ 0.58; single-fixation durations, *F*_1(5, 140)_ = 79.30, *p* < 0.001 $\eta_{p}^{2}=$ 0.75, *F*_2(5, 590)_ = 52.90, *p* < 0.001, $\eta_{p}^{2}\;=$ 0.59; gaze durations, *F*_1(5, 140)_ = 54.01, *p* < 0.001, $\eta_{p}^{2}=$ 0.67, *F*_2(5, 590)_ = 111.18, *p* < 0.001, $\eta_{p}^{2}=$ 0.54; total reading times, *F*_1(5, 140)_ = 29.31, *p* < 0.001, $\eta_{p}^{2}=$ 0.51, *F*_2(5, 590)_ = 110.57, *p* < 0.001, $\eta_{p}^{2}=$ 0.48; regressions out, *F*_1(5, 140)_ = 3.96, *p* < 0.01, $\eta_{p}^{2}=$ 0.12, *F*_2(5, 590)_ = 5.26, *p* < 0.001, $\eta_{p}^{2}=$ 0.04], although only reliable by participants for regressions-in, *F*_1(5, 140)_ = 2.80, *p* < 0.02, $\eta_{p}^{2}=$ 0.09, *F*_2(5, 590)_ = 1.85, *p* = 0.10, $\eta_{p}^{2}=$ 0.02. Compared to normal text displays, each filtered display produced more and longer fixations (*p*s < 0.001). For filtered displays, the number of fixations was lowest and fixation times shortest for medium and high spatial frequencies. The number of fixations and fixation times were both greater for very high spatial frequencies, greater still for low spatial frequencies, and greatest of all for very low spatial frequencies (*p*s < 0.01). All filtered displays except very low spatial frequencies produced lower than normal word-skipping rates and, for filtered displays, word-skipping rates were highest for very low spatial frequencies, and equally lower for low, medium, high, and very high spatial frequencies (all *p*s < 0.05). Finally, more than normal regressions in were made for low spatial frequencies only, and more than normal regressions out were made for very low and low spatial frequencies only (*p*s < 0.05).

In addition to these main effects, interactions between reading ability and display condition were significant for single-fixation duration, *F*_1(5, 140)_ = 2.24, *p* = 0.05, $\eta_{p}^{2}=$ 0.08, *F*_2(5, 590)_ = 4.68, *p* < 0.001, $\eta_{p}^{2}=$ 0.11, gaze duration *F*_1(5, 140)_ = 2.40, *p* < 0.05, $\eta_{p}^{2}=$ 0.08, *F*_2(5, 590)_ = 3.64, *p* < 0.01, $\eta_{p}^{2}=$ 0.04, and total reading time *F*_1(5, 140)_ = 3.59, *p* < 0.01, $\eta_{p}^{2}=$ 0.11, *F*_2(5, 590)_ = 15.38, *p* < 0.001, $\eta_{p}^{2}=$ 0.12, and reliable across participants for first-fixation duration, *F*_1(5, 140)_ = 2.51, *p* < 0.05, $\eta_{p}^{2}=$ 0.09, *F*_2_ < 1. No other interactions were significant, indicating that the main effects of reading ability and display type observed for other measures did not vary with the additional factor. For single-fixation duration, gaze duration, and total reading time, fast readers produced shorter times than slow readers for all display conditions (*p*s < 0.01) and a similar finding was obtained for first-fixation duration (*p* < 0.01). Further comparisons showed that these advantages for fast readers were of similar size for, medium, high, and very high spatial frequencies, but larger for very low and low (*p*s < 0.05).

A main effect of lexical frequency was significant for word-skipping, *F*_1(1, 28)_ = 15.56, *p* < 0.001, $\eta_{p}^{2}=$ 0.36, *F*_2(1, 118)_ = 6.15, *p* < 0.02, $\eta_{p}^{2}=$ 0.05, first-fixation duration, *F*_1(1, 28)_ = 15.69, *p* < 0.001, $\eta_{p}^{2}=$ 0.37, *F*_2(1, 118)_ = 25.64, *p* < 0.001, $\eta_{p}^{2}=$ 0.21, first-pass fixations, *F*_1(1, 28)_ = 24.45, *p* < 0.001, $\eta_{p}^{2}=$ 0.46, *F*_2(1, 118)_ = 11.47, *p* < 0.01, $\eta_{p}^{2}=$ 0.11, single-fixation duration, *F*_1(1, 28)_ = 11.79, *p* < 0.01, $\eta_{p}^{2}=$ 0.30, *F*_2(1, 118)_ = 11.80, *p* < 0.01, $\eta_{p}^{2}=$ 0.24, gaze duration, *F*_1(1, 28)_ = 37.94, *p* < 0.001, $\eta_{p}^{2}=$ 0.58, *F*_2(1, 118)_ = 40.59, *p* < 0.001, $\eta_{p}^{2}=$ 0.30, total reading time, *F*_1(1, 28)_ = 50.67, *p* < 0.001, $\eta_{p}^{2}=$ 0.64, *F*_2(1, 118)_ = 39.24, *p* < 0.001, $\eta_{p}^{2}=$ 0.25, and regressions in, *F*_1(1, 28)_ = 11.62, *p* < 0.01, $\eta_{p}^{2}=$ 0.29, *F*_2(1, 118)_ = 10.26, *p* < 0.01, $\eta_{p}^{2}=$ 0.08. There was no effect of word frequency for regressions out (*F* < 1.3). Crucially, there were no interactions between reading ability and word frequency (all *F*s < 1), indicating that fast and slow readers produced similar word frequency effects. Readers skipped low lexical frequency words less often than high lexical frequency words (low lexical frequency = 7.1%, high lexical frequency = 10.1%), and produced more fixations, longer fixations, longer reading times, and more regressions in for low than high lexical frequency words (first-pass fixations, low frequency = 1.4 fixations, high frequency = 1.2 fixations; first-fixation duration, low frequency = 307 ms, high frequency = 285 ms; single-fixation duration, low frequency = 318 ms, high frequency = 295 ms; gaze duration, low frequency = 419 ms, high frequency = 348 ms; total reading time, low frequency = 641 ms, high frequency = 503 ms; regressions in, low frequency = 31.3, high frequency = 25.9).

Finally, an interaction between display condition and lexical frequency was observed for total reading times, *F*_1(5, 140)_ = 13.17, *p* < 0.001, $\eta_{p}^{2}=$ 0.32, *F*_2(5, 590)_ = 10.06, *p* < 0.001, $\eta_{p}^{2}=$ 0.08 and three other measures were reliable across participants: first-pass fixations, *F*_1(5, 140)_ = 2.44, *p* < 0.05, $\eta_{p}^{2}=$ 0.08; *F*_2_ < 1; first-fixation durations, *F*_1(5, 140)_ = 2.74, *p* < 0.05, $\eta_{p}^{2}=$ 0.10, *F*_2_ < 1; gaze durations, *F*_1(5, 140)_ = 4.43, *p* < 0.01, $\eta_{p}^{2}=$ 0.14, *F*_2_ < 2.0. No other measures showed an interaction, *F*s < 1.7. Lexical frequency effects were obtained for total reading times for all displays (*p*s < 0.05) except very low spatial frequencies. Further comparisons showed that, apart from very low spatial frequencies, lexical frequency effects on total reading times were larger than normal for all filtered displays (all *p*s < 0.01) and were equal in size for medium, high and very high spatial frequencies, but larger for low spatial frequencies (all *p*s < 0.05). No other effects were obtained (*F*s < 2.5, *p*s > 0.05).

## Discussion

The first indication to emerge from these findings is that differences between fast and slow readers were evident for target words across a broad range of eye movement measures, even when text was presented as normal, indicating that differences in eye movement behavior are a fundamental component of differences in the ability of skilled readers to identify words in text. Of particular importance is that, compared to fast readers, slow readers skipped words less often, made more, and longer fixations on words, and made more regressive saccades. More and longer fixations are associated with increased difficulty in processing words, while higher rates of regressive saccades often reflect efforts to repair processing failures, including those caused by errors of word identification or miscomprehension (e.g., Reichle et al., 2009). More and longer fixations on words and more regressions by slow readers in the present study, therefore, are consistent with slow readers experiencing generally greater reading difficulty. In contrast, fast readers were more likely to skip words, with no apparent loss of comprehension, and higher word-skipping rates combined with normal comprehension are generally taken to show that readers are better able to anticipate the identities of upcoming words in text (e.g., Schotter et al., 2012). Differences between the reading performances shown by fast and slow readers, therefore, suggest that fast readers may benefit from using more efficient predictive processes to gain a head-start in processing upcoming words (e.g., Long et al., 1994; Murray and Burke, 2003; Hersch and Andrews, 2012; Frömer et al., 2015; Hawelka et al., 2015).

But the major focus of the current study is that a substantial component of differences between fast and slow readers may be that fast readers are better able to use the spatial frequencies present in words to gain more rapid bottom-up access to lexical representations when reading. Jordan et al. (2016a) observed overall sentence differences in the use of spatial frequencies by fast and slow readers that are relevant to this distinction in the use of bottom-up visual information, and showed that fast readers make more efficient use of a broad range of spatial frequencies, and especially those that are lower. But this previous research focused on reading times and eye movements across whole sentences, and so the role of spatial frequencies in accessing lexical representations by fast and slow readers remained to be confirmed. From the findings reported in the present study, all spatial frequencies produced more and longer fixations on targets, compared to normal displays, indicating that no single band of spatial frequencies provided all the information required for the normal processing of words. However, reading behavior for both fast and slow readers was closest to normal for medium and high spatial frequency displays, and reading still occurred, albeit less efficiently, for all other spatial frequencies. Therefore, while the processing of target words was most effective for spatial frequencies (medium and high) that could convey relatively detailed visual cues about the form and location of letters in words, the processing of words was still supported when only higher spatial frequencies that provide very fine detail, or lower spatial frequencies that provide more coarse-scale cues to word identities, were present. The indication, therefore, is that a broad range of spatial frequencies can contribute to the processing of words during reading, for fast and slow skilled adult readers.

There were, however, important differences in the use of spatial frequencies by fast and slow readers. Fast readers made fewer and shorter fixations on target words than slow readers for all types of display. However, while this advantage for fast readers was similar across all spatial frequencies for several measures (word-skipping, first-pass fixations, regressions out, and regressions in), single-fixation duration, gaze duration, total reading time (and to an extent first-fixation duration) showed that these advantages for fast readers were similar in size for medium, high and very high spatial frequencies, but larger for very low and low. Fast readers therefore appeared to use all spatial frequencies more effectively than slow readers when processing words and this advantage was greatest for lower spatial frequencies during the early stages of processing words. This pattern of effects is similar to that reported by Jordan et al. (2016a) for overall sentence reading and this similarity is consistent with the widely argued opinion that word identification is the engine that drives the forward movement of the eyes (e.g., Rayner et al., 1996; Reichle et al., 1998, 2003), and so differences in the efficiency of word identification are likely to be reflected in overall sentence reading times. However, the present findings also revealed that differences in the use of spatial frequencies by fast and slow readers emerge early during the processing of words and so are likely to affect the fundamental analyses of visual input that contribute to lexical access and word identification. Indeed, the particular effectiveness of medium and high spatial frequencies for both reading abilities suggests that, for fast and slow readers, processes of word identification benefited directly from relatively detailed analyses of text that enable perception of the precise form and location of individual letters in words. Such effects are consistent with some views concerning the role of letter information in reading (e.g., Pelli et al., 2003; Davis, 2010) and with the lexical quality hypothesis which proposes that fast reading benefits from high-quality lexical representations in which orthographic information defining a particular word is stored precisely (e.g., Perfetti, 1992, 2007; Andrews, 2008, 2012).

But although lower spatial frequencies were generally less effective than other spatial frequencies for reading, their relative usefulness across reading abilities indicates an important distinction between the ways in which fast and slow readers process words. Indeed, compared to slow readers, it appears that fast readers were especially able to use lower spatial frequencies to achieve lexical access, and this may provide an effective bottom-up basis for influences of predictability that many consider to be an important component of fast reading (e.g., Long et al., 1994; Murray and Burke, 2003; Hersch and Andrews, 2012; Hawelka et al., 2015). Such a possibility also resonates with arguments that lower spatial frequencies reach cortical areas before higher spatial frequencies via magnocellular pathways (e.g., Patching and Jordan, 2005a,b; Allen et al., 2009; Jordan et al., 2012, 2014) and so fast readers may use coarse-scale parafoveal cues to pre-process the identities of upcoming words, and this is also consistent with the present finding that fast readers skip words more often. Moreover, if coarse-scale information provided by lower spatial frequencies when text is presented normally is sometimes insufficient to access the correct lexical representation, fast readers may be more able to combine the rapid processing of a word's lower spatial frequencies with contextual cues to provide top-down predictions of the word's identity, and (if necessary) also integrate this information with more detailed visual input provided by slower parvocellular pathways. Moreover, in contrast to higher spatial frequencies, lower spatial frequencies can be encoded from a range of locations (foveal, parafoveal, peripheral) relative to the point of each fixation during reading (e.g., Jordan et al., 2012; Paterson et al., 2013a,b; see also Jordan et al., 2013, 2016b), and so more effective use of lower spatial frequencies by fast readers may indicate a particularly pragmatic and useful response to the nature of human visual input and the complex demands of the process of reading.

But although the present findings point to important differences in the use of spatial frequencies by fast and slow readers to gain access to lexical representations during reading, fast and slow readers produced similar lexical frequency effects, both when text was shown normally and when text was filtered to contain only selected spatial frequencies. Indeed, fast and slow readers both produced typical lexical frequency effects, such that high lexical frequency words received fewer and shorter fixations (and were more likely to be skipped) than low lexical frequency words (e.g., Inhoff and Rayner, 1986; Rayner and Duffy, 1986; Rayner et al., 1996; Juhasz and Rayner, 2003; Juhasz et al., 2006; Paterson and Jordan, 2010), and these differences were similar for each reading ability. These findings, therefore, suggest that visual access to lexical representations is generally more efficient for fast readers than for slow, for both low and high lexical frequency words, but the relative efficiency with which low and high lexical frequency words are recognized is similar for both reading abilities. It is worth pointing out that previous research has shown that word frequency effects are smaller for skilled compared to average readers, but only when words are highly predictable in the sentence context (Ashby et al., 2005). The sentence and target word combinations used in the present research were selected intentionally so that high and low frequency words were equally unpredictable, and so broadly equivalent effects of word frequency for fast and slow readers in the present analyses may reflect this controlled lack of predictability. It will be important for future research to explore the role of predictability in more detail and to establish if word frequency effects differ for fast and slow skilled readers when words are more predictable, as this may shed further light on the ability of fast readers to more efficiently combine the processing of bottom-up visual cues (especially those provided by coarse-scale information) with contextual expectations to facilitate word recognition during reading.

Filtered displays also showed word frequency effects for each reading ability with only partial evidence of influences of display type, indicating that a range of spatial frequencies can support normal processes of word identification by fast and slow skilled adult readers. Indeed, where a reliable interaction was observed (for total reading times), word frequency effects were obtained for all displays except very low spatial frequencies. Consequently, the finding that even low spatial frequencies produced a standard word frequency effect, and that this was larger compared to higher spatial frequency displays, supports the view that detailed visual cues (e.g., those provided by medium and high spatial frequencies) are not a pre-requisite for word recognition, and that more coarse-scale cues to word identity make an important contribution (e.g., Jordan, 1990, 1995; Patching and Jordan, 2005a,b; Allen et al., 2009). Indeed, while the absence of word frequency effects when text contained only very low spatial frequencies might appear to indicate a failure of word recognition, it is unlikely that word recognition was entirely prevented as even very low spatial frequencies produced above chance performance with comprehension questions. Moreover, our approach to investigate fast and slow skilled adult reading intentionally used a technique in which the effectiveness of different spatial frequency bands in text was determined individually to avoid contamination from other spatial frequencies. But all spatial frequencies present in text are normally present simultaneously and so may integrate to exert influences on word recognition either in parallel or in close succession during reading. Under these conditions, therefore, even spatial frequencies that alone provide relatively weak contributions to word recognition may provide crucial influences on the effectiveness of other spatial frequencies when normally reading text.

In sum, the indication from the findings reported here is that a broad range of spatial frequencies contribute to the identification of words during reading by fast and slow skilled adult readers, but this use of spatial frequencies is most effective for fast readers than for slow. These findings add to the view that reading relies fundamentally on the careful orchestration of visual cues provided by a broad range of spatial frequencies present in visual sensory input (e.g., Jordan, 1990, 1995; Patching and Jordan, 2005a,b; Allen et al., 2009), and that differences in the use of these visual cues underpin variation in reading performance even in the skilled adult reading population. But, crucially, these new findings indicate that fast readers make more effective use of these spatial frequencies, and especially those that are lower, to facilitate the early processing of words, and this, combined with more efficient predictive processes, may be an essential feature of their reading advantage.

## Author contributions

TJ, KP, VM, and JD designed the experiment. TJ and KP wrote the manuscript. VM and SK designed the stimuli and implemented the experiment. JD collected the data. JD, VM, and KP analyzed the data.

## Funding

The research was funded by a research awarded to TJ and KP by the Ulverscroft Foundation, an Experimental Psychology Society Undergraduate Research Bursary awarded to JD, and an ESRC Future Research Leaders Postdoctoral Fellowship to VM.

### Conflict of interest statement

The authors declare that the research was conducted in the absence of any commercial or financial relationships that could be construed as a potential conflict of interest.
